# Respiratory Cycle Influence on Lumbosacral Muscle Function: A Tensiomyographic Analysis

**DOI:** 10.3390/muscles5020030

**Published:** 2026-04-28

**Authors:** Anthony B. Trombetta, William J. Hanney, Abigail W. Anderson, Morey J. Kolber

**Affiliations:** 1 Institute of Exercise Physiology and Rehabilitation Sciences, University of Central Florida, Orlando, FL 32816, USA; troanth@gmail.com (A.B.T.); william.j.hanney@ucf.edu (W.J.H.); abigail.wilson@ucf.edu (A.W.A.); 2 Department of Physical Therapy, Nova Southeastern University, Fort Lauderdale, FL 33328, USA

**Keywords:** erector spinae, latissimus dorsi, muscle contractile characteristics, noninvasive assessment, respiratory biomechanics, skeletal muscle stiffness, tensiomyography, trunk muscle function

## Abstract

Background: Tensiomyography (TMG) offers a noninvasive means of evaluating skeletal muscle contractile properties, including muscle displacement (Dm), delay time (Td), contraction time (Tc), half-relaxation time (Tr), and sustain time (Ts). When applied to lumbosacral musculature, interpretation may be influenced by changes in muscle stiffness that occur across the respiratory cycle. Understanding these fluctuations is essential for improving measurement consistency and data interpretation. Methods: Thirty healthy young adults (mean ± SD age = 21.07 ± 1.55 years) underwent TMG assessment of the erector spinae (ES) and latissimus dorsi (LD) at four distinct lung volumes: end-tidal inspiratory volume (ETIV), end-tidal expiratory volume (ETEV), total lung capacity (TLC), and residual volume (RV). Visual cues were used to guide participants’ respiratory phases. Paired-samples *t*-tests compared TMG parameters across respiratory conditions. Results: For the ES, significant differences were observed in Dm, Tr, and Ts between ETIV and ETEV (*p* ≤ 0.05), ETIV and TLC (*p* ≤ 0.05), and ETEV and RV (*p* ≤ 0.05). No statistically significant differences were identified for the LD (*p* ≥ 0.12). Conclusions: Some erector spinae contractile properties vary across the respiratory cycle, which may affect TMG outcomes. The findings of this research lend belief to the idea that a standardized respiratory phase during data collection may improve the reliability and comparability of TMG measurements involving trunk musculature. Future research could address the negative findings for latissimus dorsi and further determine which muscles require respiratory standardization.

## 1. Introduction

Assessment of muscle contractile properties encompasses a range of methodological approaches. Such approaches utilize diverse technologies to investigate muscle function across different contexts. Surface electromyography (sEMG), muscle torque production, shear wave ultrasound elastography, and mechanomyographic (MMG) methods have traditionally served as vital tools to examine mechanical muscle properties and muscle behaviors.

These methods have significantly advanced comprehension of muscle function. However, disadvantages among these methods are abundant. Challenges of the aforementioned methodologies include, but are not limited to, complexity, high variability, and invasiveness. Moreover, these methods are costly and limited in applicability beyond laboratory environments.

A newer MMG method called tensiomyography (TMG) has demonstrated significant potential in the examination of muscle functionality while evading the limitations of other methods for muscle function measuring. TMG is a noninvasive, portable measurement tool that allows for the analysis of the contractile and mechanical properties of muscles [1,2]. Current research reveals the various applications of TMG in exercise testing, training, and healthcare settings [3]. Additionally, TMG has potential as a valuable tool for screening, diagnosing, and monitoring responses to surgical treatments [4].

**Figure 1:**
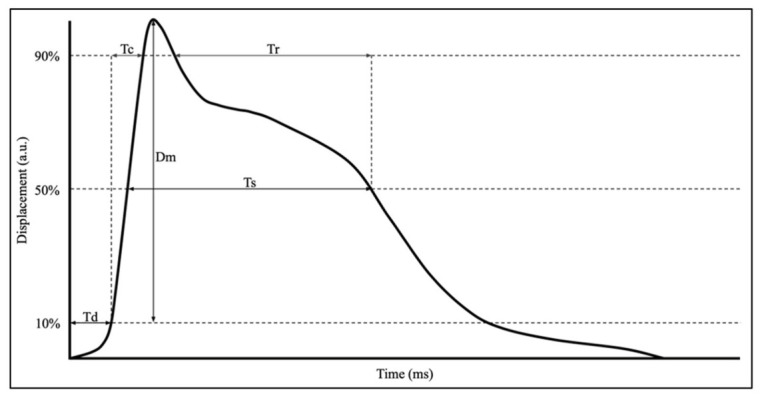
Illustration of a classic wave produced by TMG with all five parameters labeled.

The advantages of TMG over other MMG methods and other muscle analysis devices lie in its ability to control the pre-tension of the sensor, which positively influences the evaluation of contraction dynamics [5]. The device utilizes a high-precision displacement sensor, positioned perpendicular to the muscle belly’s surface with a pre-tension of 0.2 N/cm^2^ [6]. This sensor can evaluate various parameters derived from the waveform generated following percutaneous neuromuscular stimulation, ranging from submaximal to maximal levels [3]. Five main parameters are acquired: a spatial measurement, measured in millimeters (mm); and four temporal measurements, measured in milliseconds (ms) [6]. These measurements are extracted from the twitch displacement–time curve created and include muscle displacement (Dm), delay time (Td), contraction time (Tc), half-relaxation time (Tr), and sustain time (Ts) [6]. Of these parameters, the most evaluated and with the highest level of reliability are Dm, Td, and Tc [3,5,7]. (See Figure 1).

Dm is representative of the maximal radial displacement of the muscle belly [3,8]. Td illustrates the amount of time for the muscle to reach 10% of the observed displacement [3]. Tc demonstrates the time from 10% of the measured Dm to 90% of the measured Dm [3].

Consistent measurements with minimal fluctuations (normalized mean errors ranging from 0.5% to 2%) can be achieved through consecutive measurements, provided there is adequate time between measurements (10 s) and consistent initial pressure is maintained [5]. Initial pressure can be retained by adjusting and repositioning the displacement transducer after each attempt [5]. To achieve the highest individual response, stimulation should be gradually intensified until the maximum radial displacement amplitude for the individual is reached [6].

Tensiomyography offers quick and precise diagnostic information about lumbosacral muscle function without causing discomfort or interrupting personal schedules [9]. Erector spinae and rectus abdominis muscles can be measured with little challenge as TMG can be conducted noninvasively while a participant lies prone and stable [9]. However, it is known that muscle atrophy increases Dm, while an increase in the tension, tone, or stiffness of muscles decreases Dm [10,11].

Despite the benefits of TMG over alternative methods, certain challenges persist in the analysis of lumbosacral muscle function. Primarily, difficulties arise from the fact that a participant must lie prone, allowing for access to the lumbosacral muscles. In this position, lumbosacral muscles undergo continuous movement and fluctuations in contractility throughout the respiratory cycle [12]. It has been noted that the diaphragm, paraspinal muscles, and abdominal muscles undergo distinct modulation during the respiratory cycle [12]. An increase in stiffness occurs during tasks in which lung volume is held above (total lung capacity) and below (residual volume) one’s normal tidal volume—the normal respiration cycle (end-tidal inspiratory volume, end-tidal expiratory volume) [12]. There is no significant change in stiffness over tidal volume [12]. Amid heightened respiratory exertion, lumbosacral muscles show elevated activity [12]. Notably, a more pronounced difference in stiffness was observed during expiration, with maximal expiration showing the greatest increase in stiffness [12]. Therefore, it is hypothesized that, for all TMG parameters, heightened levels of respiration will produce drastically different data when compared to tidal levels of respiration.

These confounding variables pose potential variability in the measurements obtained via TMG, as heightened muscle contraction may affect the parameters and yield misleading readings. In their meta-analysis search, Lohr et al. reviewed 49 research articles and found only one involving the erector spinae, a lumbosacral muscle [5]. The latissimus dorsi was selected as a comparison muscle due to its anatomical connections to the thoracodorsal fascia, which is also a key structure involved in respiratory mechanics. This fascia serves as an attachment site for several muscles that contribute to trunk and respiratory function, suggesting that latissimus dorsi may exhibit activity patterns influenced by respiration. In contrast, the erector spinae has a more direct role in spinal extension and stabilization, which may lead to different modulation during respiratory phases. This anatomical distinction provides a physiological rationale for expecting divergent respiratory effects between the two muscles. To date, a standardized protocol for testing procedures to mitigate these confounders has not been established. A noticeable gap in research exists for protocols pertaining to the measurement of lumbosacral muscles; therefore, this study aims to examine the variability of lumbosacral muscle function throughout the respiratory cycle as measured by TMG.

## 2. Results

Thirty healthy young adults participated in the study (10 males, 20 females; mean ± SD age = 21.07 ± 1.55 years; height = 168.06 ± 9.94 cm; body mass = 64.82 ± 14.50 kg). Twenty-eight participants were right-hand dominant and two were left-hand dominant (Table 1).

Tensiomyographic parameters of the erector spinae (ES) demonstrated significant variation across respiratory conditions (Table 2 and Table 3). Mean muscle displacement (Dm) differed significantly between end-tidal inspiratory volume (ETIV) and end-tidal expiratory volume (ETEV; difference in means = −0.779, *t*(29) = −6.22, *p* < 0.001), indicating greater displacement during expiration. Additional significant Dm differences were observed between ETIV and total lung capacity (TLC; difference in means = 0.653, *t*(29) = 2.54, *p* = 0.017), and between ETEV and both TLC (difference in means = 1.432, *t*(29) = 5.70, *p* < 0.001) and residual volume (RV; difference in means = 1.06, *t*(29) = 5.33, *p* < 0.001). Half-relaxation time (Tr) and sustain time (Ts) also differed significantly between ETIV and ETEV (difference in means = −138.95, *t*(29) = −2.77, *p* = 0.010; difference in means = −241.933, *t*(29) = −3.87, *p* < 0.001, respectively) and between ETEV and both TLC (difference in means = 119.273, 207.318, *p* = 0.024, <0.001) and RV (difference in means = 134.705, 205.683, *p* = 0.011, 0.002). No statistically significant changes were found for delay time (Td) or contraction time (Tc) across any comparisons (*p* > 0.05).

Analyses of the latissimus dorsi (LD) revealed no statistically significant differences in any TMG-derived variable across respiratory phases (Table 4 and Table 5). Comparisons for Dm, Td, Tc, Tr, and Ts yielded *p* ≥ 0.056, indicating stable contractile behavior throughout the respiratory cycle.

Overall, the ES exhibited significant fluctuations in mechanical properties, particularly in Dm, Tr, and Ts, across phases of respiration. These findings suggest that respiratory phase can influence TMG-derived measurements in paraspinal musculature. In contrast, LD contractile properties remained consistent regardless of respiratory condition.

## 3. Discussion

The aim of this study was to investigate differences in lumbosacral muscle function throughout the respiratory cycle. Focus was placed on the erector spinae and latissimus dorsi due to the perceived effects of respiration on TMG analyses. Current research using TMG focuses primarily on appendages, where muscle function does not fluctuate during respiration. However, the constant movement of certain muscles during respiration makes them of particular interest. Further, these muscles are easily accessible and able to be analyzed using TMG. The diaphragm, which is the major respiratory muscle, cannot be analyzed with TMG because of its location within the chest. Consequently, muscles that are impacted by the diaphragm when lying prone were chosen.

Of the data collected, muscle displacement of the erector spinae showed the most significant differences in values under the four respiratory conditions that were analyzed. This finding signifies that muscle analyses may be misinterpreted when using TMG as the primary method of analysis if the tester does not document the phase of respiration for measurements.

Current data shows that erector spinae and abdominal muscles function at increased levels during heightened respiratory effort [12]. The results of this study are concurrent with that data. ETIV and TLC comparisons showed significant differences in Dm measurements. ETEV and TLC comparisons, along with ETEV and RV comparisons, showed statistically significant differences in Dm, Tr, and Ts. ETIV and ETEV represented respiratory conditions of normal effort, while TLC and RV were of heightened effort. These analyses add to the understanding of respiration altering muscle function on the erector spinae. Further, analyses completed for erector spinae data showed no statistical significance for Td and Tc. This is noteworthy, as other research with TMG has illustrated that Td and Tc are among the most reliable of the parameters recorded [3,5,7]. However, most clinicians and researchers rely on Dm measurements for their analyses. The multitude of statistically significant Dm findings in this research, therefore, still shows that lumbosacral muscles function differently throughout the respiratory cycle. This ultimately affects the reliability of TMG analyses done on such muscles.

Of note is that there were no significant differences in measurements taken on the latissimus dorsi. This finding was not consistent with the original hypotheses of this study. As previously mentioned, the erector spinae has been proven to function differently under heightened respiratory conditions [12]. The same conclusions have yet to be drawn for the latissimus dorsi in the existing literature. Although this was known, the researchers hypothesized that the constant rise and fall of the diaphragm during respiration would cause TMG measurements on the latissimus dorsi to fluctuate. Further, it was thought that the latissimus dorsi would elicit differences in measurements similar to those found of the erector spinae. One possible explanation for this discrepancy is the location of each muscle and its relation to respiration. Alternative explanations—including potential measurement limitations, insufficient statistical power, or an incomplete understanding of the underlying physiological mechanism—may also account for the null results and should be explored in future research. This study does not provide any data to the counter or add to the existing literature in relation to the altered functioning of the latissimus dorsi throughout respiration.

It is noted in the existing literature that heightened inspiratory action causes a greater increase in ES activity than heightened expiratory action [12]. The hypotheses for this study were formed under the belief that the increased activity caused by each respiration condition manifested in different ways, leading to the greater increase in ES activity that has been seen in other research. Therefore, it was thought that TLC and RV data would result in measurements with statistically significant differences. However, data comparisons between TLC and RV regarding the ES showed no statistical significance. A possible reason for this hypothesis not being proven is that the increased activity of the ES under heightened inspiration and heightened expiration is not as different as previously thought. Both respiratory extremes must result in increased ES activity that is similar in nature, despite inspiration and expiration being opposites.

As this was among the first study of its kind, there are several limitations that arose. Such limitations of this research primarily relate to how the tidal respiratory conditions were observed. This study used visual cues corresponding to inhalation and exhalation for ETIV and ETEV conditions, respectively. Visual observations were employed to increase the repeatability of the procedure and to account for the limited availability of advanced respiratory monitors in typical clinical settings. This approach enhances the potential for clinicians to apply the findings without specialized equipment. The practical implications of this study lie in the use of simple, accessible methods to monitor respiratory patterns. By observing diaphragm movement directly, rather than relying on advanced monitoring tools, clinicians can employ a repeatable and feasible method to assess respiratory patterns. This approach facilitates the translation of these findings into real-world clinical practice. Additionally, there was no inter-rater or intra-rater reliability data provided for the visual determination of respiratory phases. This was also done to support the opportunity for clinical application.

Another limitation includes possible confounding variables, such as BMI (min = 18.129, mean = 22.905, max = 40.669). Trends in data collection showed a higher BMI correlated with less muscle response to electrical stimuli. Although no analyses were performed to track correlation between these factors, it is worth noting. This research did not anticipate BMI as a confounding variable; therefore, future research may wish to expand on this idea. Moreover, this experiment has a few highly skewed data points illustrated by high standard deviations in relation to their respective means, as shown in Table 2 and Table 4. For example, Tr for ES at ETEV has a standard deviation of 274.716 compared to a mean of 261.780, indicating that assumptions of parametric testing may be violated.

A further limitation is a lack of randomizing or counterbalancing for the respiratory conditions. As a result, there is possibility of data influence by order effects or participant fatigue. In addition, multiple comparisons were conducted across several parameters without formal adjustment, which may have inflated the risk of Type I error. Consequently, some statistically significant findings should be interpreted with caution, as they may not remain significant after applying conservative correction methods (e.g., Bonferroni adjustment). An additional consideration is that some observed significant effects may, in part, reflect characteristics of the measurement approach rather than true underlying physiological differences. Additionally, significant findings may have alternate explanations beyond respiratory biomechanics, such as postural adjustments, anticipatory muscle activity, or cardiovascular effects. Finally, the analyses performed utilized the highest of the two data values for each TMG parameter, rather than using the average of the two. These points suggest the findings of this research should be interpreted thoughtfully, and future studies could benefit from incorporating complementary measurement methods to further clarify the source of the observed effects.

As far as the authors are aware, this is the first study to examine how respiratory conditions may skew muscle function when examining with TMG. This data serves to illustrate that some muscles do function at altered levels throughout the respiratory cycle. Therefore, data collected using TMG on muscles that are affected by respiration may not be accurate to the true functioning of the muscle. This realization may be critical in clinical settings where TMG is used. Without accounting for these variations caused by respiration, TMG analyses may not be valid and could result in misinterpretations and misdiagnoses.

The authors acknowledge that the study’s analytical design would ideally require a larger sample size and the inclusion of structured experimental groups, such as stratification by age or gender, to strengthen generalizability. However, the sample size of 30 participants was justified through a priori power analysis, and participant gender distribution is reported in the demographic table. While these factors cannot be modified retrospectively, their acknowledgment ensures that the study’s limitations are appropriately contextualized. Moreover, several variables demonstrated large standard deviations relative to their means, suggesting potential skewness or the presence of outliers. As formal assumption testing was not conducted, the extent to which these factors influenced the results is unknown. Therefore, findings should be interpreted with caution, and future research should incorporate normality testing, outlier diagnostics, and consideration of non-parametric or robust statistical approaches.

Future research could expand on this study to create a standardized TMG protocol for analyzing muscles such as the erector spinae. Notably, this study was conducted on healthy patients who did not have difficulty breath-holding or following respiratory instructions. Future research should focus on symptomatic patient populations to allow for even greater clinical application. Doing so would ensure TMG analyses are valid, and diagnoses established are accurate. As TMG continues to be used in clinical practices, these findings are likely to help ensure proper usage and better understanding of TMG’s capabilities in healthcare. The value, in part of this study, resides in the need for clinicians and researchers to recognize the importance of documenting the phase of respiration when using data from TMG.

## 4. Materials and Methods

### 4.1. Study Design

The design of this study was that of a prospective cohort. The primary research objective was to examine the variation in lumbosacral muscle function throughout respiration. Secondary objectives included suggesting a standardized protocol for TMG procedures when measuring lumbosacral muscle function.

### 4.2. Participants

Thirty young adults, aged 18–25, were eligible to participate in the study. Participants were required to take a screening questionnaire before scheduling an appointment for data analysis. Only participants with no self-reported previous history of lumbosacral muscle disorders or any significant neurological, respiratory, or cardiovascular diseases were included. Participants were required to be fluent in reading and writing English. Participants could not be pregnant.

### 4.3. Procedures

Participants read a consent form and written consent was obtained. Demographic data (age, sex assigned at birth, self-reported dominant laterality, self-reported height and weight) was collected. All procedures occurred in a temperature- and humidity-controlled room to reduce possible effects of environmental conditions on muscle function.

Previous research has utilized pneumotachographs and oscilloscopes to measure the airflow of participants; however, this study relied on visual examination of the rise and fall of the diaphragm to measure airflow [12]. Utilizing visual cues rather than complex machinery allows for greater opportunity for the results of this study to be used by clinicians. Tidal inspiration was observed throughout interactions with participants, noting approximate changes in the height of the diaphragm. End-tidal inspiratory volume (ETIV) corresponded to visual cues that indicated a participant had inhaled the normal volume they do during respiration. End-tidal expiratory volume (ETEV) related to visual cues that showed a participant had exhaled the normal volume they would during respiration. Total lung capacity (TLC) was obtained by asking the participant to inhale as much oxygen as possible, more so than they do during tidal breathing. This volume was held for approximately three seconds while the TMG measurement was taken. Residual volume (RV) occurred when a participant was asked to expel as much air from their lungs as possible, greater than that which occurs during tidal breathing. This volume was also held for approximately three seconds for TMG analysis to occur. All respiratory maneuvers were demonstrated by the researcher to familiarize participants with the procedures and to ensure participants could achieve and hold target lung volumes.

**Figure 2:**
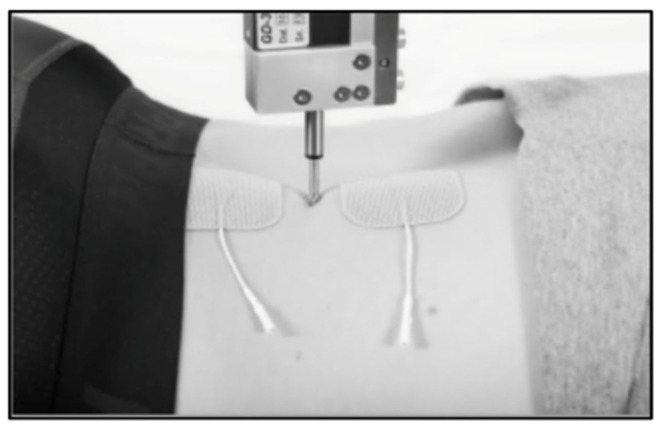
TMG set-up for erector spinae.

First, two electrodes were placed roughly 5 cm apart on the muscle belly of the participant’s self-reported dominant side [13]. The TMG displacement sensor (TMG-BMC, Ljubljana, Slovenia) was set perpendicular to the muscle belly and lowered until contact with the participant’s muscle was made. (See Figure 2). An initial amplitude of 20 mA was sent through the electrodes via an electrical stimulator (TMG-S1) [13]. A baseline measurement was recorded by progressively increasing the amplitude by 10 mA increments until the Dm response no longer increased on the TMG-OK 3.0 software. This occurred when two sequential measurements, with a difference of 10 mA, did not change the peak (Dm) of the TMG graph produced. A constant peak between two amplitudes signified the proper baseline amplitude had been achieved. This amplitude was then utilized for non-manipulated and breath-manipulated measurements. Baseline amplitudes were not reported within this research, as they were not important for our analyses.

An electrical impulse at the baseline amplitude was administered when visual cues indicated the participant had reached ETIV. The impulse was administered twice with a rest period of at least 10 s between the impulses, which has been noted by Lohr et al. and Macgregor et al. as a common inter-stimulus interval for tensiomyographic analyses [14]. The higher of the two data values was recorded and used for data analyses. The use of higher values was opted for to maximize readings. Moreover, it was important for this research to utilize values that corresponded with maximum muscle contraction to ensure appropriate analyses were completed. An electrical impulse at the baseline amplitude was administered when visual cues indicated the participant had reached ETEV. The impulse was administered twice with a rest period of at least 10 s between the impulses. The highest of the two data values was recorded and used for data analyses. To represent TLC, the participant was instructed to inhale as much air as they could and hold that air in while the measurement was taken. An electrical impulse at the baseline amplitude was administered. The impulse was administered twice with a rest period of at least 10 s between the impulses. The highest of the two data values was recorded and used for data analyses. For RV, the participant was instructed to exhale all air from their lungs and refrain from inhaling while the measurement was taken. An electrical impulse at the baseline amplitude was administered. The impulse was administered twice with a rest period of at least 10 s between the impulses. The highest of the two data values was recorded and used for data analyses.

**Figure 3:**
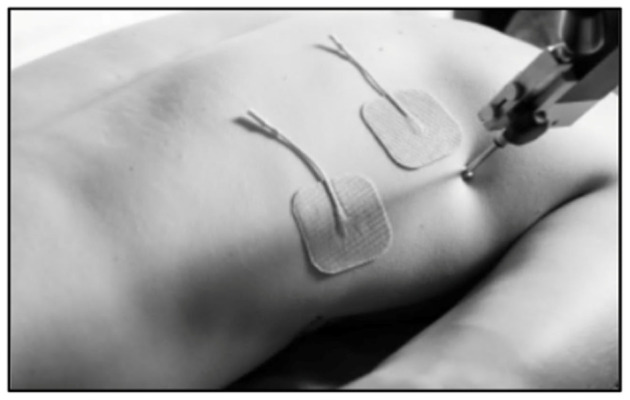
TMG set-up for latissimus dorsi.

Next, the participant was instructed to lie prone on the treatment table for access to the latissimus dorsi muscles. Two electrodes were placed on the muscle belly of the participant’s self-reported dominant laterality, with roughly 5 cm between them. The TMG sensor was set perpendicular to the muscle belly and lowered to contact the skin. (See Figure 3). Like that of the erector spinae protocol, the electrical stimulator sent an initial amplitude of 20 mA through the electrodes. A baseline measurement was found by increasing the electrical amplitude by 10 mA until the Dm response remained stagnant on the TMG-OK 3.0 software. This amplitude was utilized for all further measurements.

The baseline amplitude was used to administer an electrical impulse when visual cues indicated ETIV for a participant. The impulse was administered twice with a rest period of at least 10 s between the impulses. The highest of the two data values was recorded and used for data analyses. An electrical impulse at the participant’s baseline amplitude was administered when visual cues indicated ETEV. The impulse was administered twice with a rest period of at least 10 s between the impulses. The highest of the two data values was recorded and used for data analyses. The participants were instructed to inhale as much air as they could and hold that air in while the measurement was taken. This represented TLC. An electrical impulse at the baseline amplitude was administered. The impulse was administered twice with a rest period of at least 10 s between the impulses. The highest of the two data values was recorded and used for data analyses. The participant was asked to exhale all air from their lungs and refrain from inhaling while the measurement was taken. This represented RV. An electrical impulse at the baseline amplitude was administered. The impulse was administered twice with a rest period of at least 10 s between the impulses. The highest of the two data values was recorded and used for data analyses.

### 4.4. Statistical Analysis

An a priori power analysis was conducted for a two-tailed paired-samples *t*-test (α = 0.05), indicating that a sample size of 30 participants would provide ≥80% power to detect a moderate-to-large within-subject effect (Cohen’s d ≥ 0.55) in respiratory muscle displacement outcomes. This sample size was selected to ensure adequate power for the primary comparisons while accommodating potential variability across respiratory conditions. JASP software v0.96 (JASP Team, Amsterdam, The Netherlands) was utilized to obtain descriptive statistics for participant demographic data and Dm, Td, Tc, Tr, and Ts measurements under the four respiratory conditions. Paired-samples *t*-tests were run to compare respective TMG parameters from the four respiration conditions. The level of significance was set at *p* ≤ 0.05. Given the exploratory nature of this study and the limited sample size, no formal correction for multiple comparisons was applied. Therefore, statistical findings should be interpreted as hypothesis-generating. Assumptions of normality and the presence of outliers were not formally assessed as part of the original analytical plan. Given the within-subject design and sample size (*n* = 30), paired-samples *t*-tests were selected due to their relative robustness to moderate deviations from normality. However, substantial skewness or the presence of outliers may influence results; therefore, findings should be interpreted with caution.

## Figures and Tables

**Table 1:** Participant demographic data.

	Baseline Characteristics
Males	10
Females	20
Age in years, mean ± SD	21.067 ± 1.552
Height (cm), mean ± SD	168.063 ± 9.940
Body mass (kg), mean ± SD	64.818 ± 14.503
Body mass index, mean ± SD	22.905 ± 4.557
Handedness	
Right	28
Left	2

SD: standard deviation, cm: centimeters, kg: kilograms.

**Table 2:** Variable data for erector spinae measurements.

	ETIV	ETEV	TLC	RV
Dm, mean ± SD	2.953 ± 1.747	3.732 ± 1.695	2.300 ± 1.297	2.672 ± 1.627
Td, mean ± SD	23.841 ± 6.305	31.005 ± 38.895	30.113 ± 41.689	30.752 ± 38.337
Tc, mean ± SD	20.753 ± 7.686	24.632 ± 10.115	20.560 ± 9.645	28.935 ± 33.542
Tr, mean ± SD	122.830 ± 133.642	261.780 ± 274.716	142.507 ± 157.190	127.075 ± 102.852
Ts, mean ± SD	211.209 ± 206.525	453.142 ± 343.475	245.824 ± 222.092	247.459 ± 177.766

SD: standard deviation, Dm: maximum radial displacement, Td: delay time, Tc: contraction time, Tr: relaxation time, Ts: sustain time.

**Table 3:** Erector spinae analyses.

		Statistical Analyses						
							95% CI for Cohen’s d	
Measure 1	Measure 2	t	df	*p*	Cohen’s d	Cohen’s d SE	Lower	Upper
ETIV	ETEV							
	Dm	−6.222	29	<0.001 *	−1.136	0.093	−1.591	−0.669
	Td	−0.906	29	0.372	−0.165	0.335	−0.524	0.196
	Tc	−1.873	29	0.071	−0.342	0.236	−0.708	0.029
	Tr	−2.773	29	0.010 *	−0.506	0.238	−0.883	−0.122
	Ts	−3.870	29	<0.001 *	−0.707	0.240	−1.103	−0.301
ETIV	TLC							
	Dm	2.544	29	0.017 *	0.464	0.170	0.084	0.838
	Td	−0.830	29	0.413	0.463	0.144	0.082	0.836
	Tc	0.132	29	0.896	0.024	0.166	−0.334	0.382
	Tr	−0.501	29	0.620	−0.092	0.270	−0.449	0.268
	Ts	−0.717	29	0.479	−0.131	0.0226	−0.489	0.230
ETIV	RV							
	Dm	1.489	29	0.147	0.272	0.113	−0.095	0.634
	Td	−0.979	29	0.335	−0.179	0.256	−0.538	0.183
	Tc	−1.188	29	0.244	−0.217	0.316	−0.577	0.147
	Tr	−0.172	29	0.865	−0.031	0.205	−0.380	0.327
	Ts	−0.780	29	0.442	−0.142	0.242	−0.501	0.218
ETEV	TLC							
	Dm	5.700	29	<0.001 *	1.041	0.201	0.589	1.481
	Td	0.081	29	0.936	0.015	0.273	−0.343	0.373
	Tc	1.798	29	0.083	0.328	0.235	−0.042	0.693
	Tr	2.379	29	0.024 *	0.434	0.228	0.056	0.806
	Ts	3.810	29	<0.001 *	0.696	0.201	0.291	1.091
ETEV	RV							
	Dm	5.326	29	<0.001 *	0.972	0.145	0.531	1.403
	Td	0.025	29	0.980	0.005	0.258	−0.353	0.362
	Tc	−0.853	29	0.980	−0.156	0.146	−0.515	0.206
	Tr	2.734	29	0.011 *	0.499	0.240	0.115	0.875
	Ts	3.375	29	0.002 *	0.616	0.234	0.221	1.003
TLC	RV							
	Dm	−1.562	29	0.129	−0.285	0.162	−0.648	0.082
	Td	−0.458	29	0.650	−0.084	0.031	−0.441	0.276
	Tc	−1.243	29	0.224	−0.227	0.289	−0.588	0.137
	Tr	0.450	29	0.656	0.082	0.259	−0.277	0.440
	Ts	−0.039	29	0.969	−0.007	0.205	−0.365	0.351

ETIV: end-tidal inspiratory volume, ETEV: end-tidal expiratory volume, TLC: total lung capacity, RV: residual volume, Dm: maximum radial displacement, Td: delay time, Tc: contraction time, Tr: relaxation time, Ts: sustain time, CI: confidence interval, SE: standard error. * Denotes statistical significance.

**Table 4:** Variable data for latissimus dorsi measurements.

	ETIV	ETEV	TLC	RV
Dm, mean ± SD	4.424 ± 2.272	4.351 ± 2.239	3.994 ± 2.004	4.562 ± 2.554
Td, mean ± SD	25.408 ± 4.997	26.755 ± 8.969	26.384 ± 9.970	25.089 ± 3.991
Tc, mean ± SD	39.017 ± 20.126	37.609 ± 14.702	38.754 ± 19.862	38.071 ± 16.330
Tr, mean ± SD	76.587 ± 54.341	138.773 ± 191.946	109.851 ± 107.223	99.719 ± 76.630
Ts, mean ± SD	220.284 ± 96.223	301.464 ± 263.735	271.305 ± 187.911	233.966 ± 111.077

SD: standard deviation, Dm: maximum radial displacement, Td: delay time, Tc: contraction time, Tr: relaxation time, Ts: sustain time.

**Table 5:** Latissimus dorsi analyses.

		Statistical Analyses						
							95% CI for Cohen’s d	
Measure 1	Measure 2	t	df	*p*	Cohen’s d	Cohen’s d SE	Lower	Upper
ETIV	ETEV							
	Dm	0.502	29	0.619	0.092	0.065	−0.268	0.450
	Td	−1.051	29	0.302	−0.192	0/159	−0.551	0.171
	Tc	0.585	29	0.563	0.107	0.128	−0.253	0.465
	Tr	−1.709	29	0.098	−0.312	0.264	−0.676	0.057
	Ts	−1.994	29	0.056	−0.364	0.174	−0.731	0.009
ETIV	TLC							
	Dm	1.562	29	0.129	0.285	0.130	−0.082	0.648
	Td	−0.655	29	0.517	−0.120	0.168	−0.478	0.241
	Tc	0.079	29	0.938	0.014	0.167	−0.344	0.372
	Tr	−1.504	29	0.143	−0.275	0.266	−0.637	0.092
	Ts	−1.941	29	0.062	−0.354	0.155	−0.721	0.018
ETIV	RV							
	Dm	−0.422	29	0.676	−0.077	0.134	−0.435	0.282
	Td	0.466	29	0.644	0.085	0.148	−0.274	0.443
	Tc	0.468	29	0.643	0.085	0.105	−0.274	0.443
	Tr	−1.675	29	0.105	−0.306	0.209	−0.670	0.063
	Ts	−0.591	29	0.559	−0.108	0.223	−0.466	0.252
ETEV	TLC							
	Dm	1.633	29	0.113	0.298	0.104	−0.070	0.662
	Td	0.785	29	0.439	0.143	0.046	−0.218	0.502
	Tc	−0.432	29	0.669	−0.079	0.145	−0.437	0.280
	Tr	0.958	29	0.346	0.175	0.182	−0.187	0.534
	Ts	0.929	29	0.361	0.170	0.133	−0.192	0.529
ETEV	RV							
	Dm	−0.677	29	0.504	−0.124	0.128	−0.482	0.237
	Td	1.169	29	0.252	0.213	0.186	−0.150	0.574
	Tc	−0.328	29	0.745	−0.060	0.089	−0.417	0.299
	Tr	0.996	29	0.328	0.182	0.275	−0.181	0.541
	Ts	1.330	29	0.194	0.243	0.252	−0.122	0.604
TLC	RV							
	Dm	−1.836	29	0.077	−0.335	0.133	−0.700	0.036
	Td	0.780	29	0.442	0.142	0.199	−0.219	0.501
	Tc	0.275	29	0.785	0.050	0.133	−0.308	0.408
	Tr	0.457	29	0.651	0.083	0.237	−0.276	0.441
	Ts	1.029	29	0.312	0.188	0.234	−0.175	0.547

ETIV: end-tidal inspiratory volume, ETEV: end-tidal expiratory volume, TLC: total lung capacity, RV: residual volume, Dm: maximum radial displacement, Td: delay time, Tc: contraction time, Tr: relaxation time, Ts: sustain time, CI: confidence interval, SE: standard error.

## Data Availability

The original contributions presented in this study are included in the article. Further inquiries can be directed to the corresponding author.

## References

[1] Lohr C., Braumann K.-M., Reer R., Schroeder J., Schmidt T. (2018). Reliability of Tensiomyography and Myotonometry in Detecting Mechanical and Contractile Characteristics of the Lumbar Erector Spinae in Healthy Volunteers. Eur. J. Appl. Physiol..

[2] García-Sillero M., Benítez-Porres J., García-Romero J., Bonilla D.A., Petro J.L., Vargas-Molina S. (2021). Comparison of Interventional Strategies to Improve Recovery after Eccentric Exercise-Induced Muscle Fatigue. Int. J. Environ. Res. Public Health.

[3] Martín-Rodríguez S., Loturco I., Hunter A.M., Rodríguez-Ruiz D., Munguia-Izquierdo D. (2017). Reliability and Measurement Error of Tensiomyography to Assess Mechanical Muscle Function: A Systematic Review. J. Strength Cond. Res..

[4] Martín-Rodríguez S., Alentorn-Geli E., Tous-Fajardo J., Samuelsson K., Marín M., Álvarez-Díaz P., Cugat R. (2017). Is Tensiomyography a Useful Assessment Tool in Sports Medicine?. Knee Surg. Sports Traumatol. Arthrosc..

[5] Krizaj D., Simunic B., Zagar T. (2008). Short-Term Repeatability of Parameters Extracted from Radial Displacement of Muscle Belly. J. Electromyogr. Kinesiol..

[6] Lohr C., Schmidt T., Medina-Porqueres I., Braumann K.-M., Reer R., Porthun J. (2019). Diagnostic Accuracy, Validity, and Reliability of Tensiomyography to Assess Muscle Function and Exercise-Induced Fatigue in Healthy Participants. A Systematic Review with Meta-Analysis. J. Electromyogr. Kinesiol..

[7] Tous-Fajardo J., Moras G., Rodríguez-Jiménez S., Usach R., Doutres D.M., Maffiuletti N.A. (2010). Inter-Rater Reliability of Muscle Contractile Property Measurements Using Non-Invasive Tensiomyography. J. Electromyogr. Kinesiol..

[8] Simunič B. (2012). Between-Day Reliability of a Method for Non-Invasive Estimation of Muscle Composition. J. Electromyogr. Kinesiol..

[9] Park S. (2020). Theory and Usage of Tensiomyography and the Analysis Method for the Patient with Low Back Pain. J. Exerc. Rehabil..

[10] Pisot R., Narici M.V., Simunic B., De Boer M., Seynnes O., Jurdana M., Biolo G., Mekjavić I.B. (2008). Whole Muscle Contractile Parameters and Thickness Loss during 35-Day Bed Rest. Eur. J. Appl. Physiol..

[11] Hunter A.M., Galloway S.D.R., Smith I.J., Tallent J., Ditroilo M., Fairweather M.M., Howatson G. (2012). Assessment of Eccentric Exercise-Induced Muscle Damage of the Elbow Flexors by Tensiomyography. J. Electromyogr. Kinesiol..

[12] Shirley D., Hodges P.W., Eriksson A.E.M., Gandevia S.C. (2003). Spinal Stiffness Changes throughout the Respiratory Cycle. J. Appl. Physiol..

[13] Kalc M., Puš K., Paravlic A., Urbanc J., Šimunič B. (2023). Diagnostic Accuracy of Tensiomyography Parameters for Monitoring Peripheral Neuromuscular Fatigue. J. Electromyogr. Kinesiol..

[14] Macgregor L.J., Ditroilo M., Smith I.J., Fairweather M.M., Hunter A.M. (2016). Reduced Radial Displacement of the Gastrocnemius Medialis Muscle After Electrically Elicited Fatigue. J. Sport Rehabil..

